# A Dilemma in Staging of Esophageal Cancer: How Should We Stage ypT0 N2 M0 Esophageal Cancer after Neoadjuvant Therapy?

**DOI:** 10.1155/2015/158626

**Published:** 2015-08-06

**Authors:** Sebahattin Celik, Remzi Erten, Abdulsamed Batur, Burak Suvak

**Affiliations:** ^1^Department of General Surgery, Yüzüncü Yıl University, 65200 Van, Turkey; ^2^Department of Pathology, Yüzüncü Yıl University, 65200 Van, Turkey; ^3^Department of Radiology, Yüzüncü Yıl University, 65200 Van, Turkey; ^4^Department of Gastroenterology, Yüzüncü Yıl University, 65200 Van, Turkey

## Abstract

*Background*. Since neoadjuvant treatment in esophageal cancer began to become popular, a complete pathological response at the primary tumour site has been commonly reported. An issue of conflict is whether complete response in the esophageal lumen means that the esophagus is completely tumour-free. Another important issue is whether lymph nodes that are retrieved from pathologically complete response cases are also tumour-free or not. There is a gap in the esophageal cancer staging system for ypT0 N2 M0 tumours that have received neoadjuvant therapy. Here, we will discuss the problem about staging of esophageal cancer associated with neoadjuvant therapy. *Case*. A female aged 40 years complaining of dysphagia was diagnosed as having locally advanced thoracic esophageal cancer. Neoadjuvant therapy decision was taken by oncology committee. Six weeks after neoadjuvant therapy, with a curative intention, minimal invasive surgery was performed. The pathology report was as follows. “There were no neoplastic cells in the suspected area of the esophageal mucosa upon examination with all staining. There was no cancer at resection margins. Four metastatic lymph nodes were infiltrated with squamous cell cancer.” *Conclusion*. Despite the growing use of neoadjuvant treatment in locally advanced esophageal cancer in world, we do not have a protocol for the evaluation of these patients' pathology reports. We believe that new studies and new ideas are needed to resolve this dilemma associated with neoadjuvant therapy.

## 1. Scientific Background

There is no question that the presence of lymph node (LN) metastasis in esophageal cancer is one of the most powerful prognostic indicators. Esophageal cancer staging is defined by the American Joint Committee on Cancer (AJCC) Staging System, which establishes tumor-node-metastasis (TNM) subclassifications based on the depth of invasion of the primary tumor (T), lymph node involvement (N), and extent of metastatic disease (M). The most recent 7th edition of the AJCC Cancer Staging Manual for esophageal and esophagogastric junction cancers was developed based on a database of 4627 esophagectomy patients who were not treated with induction or adjuvant therapy [1]. Because of the poor prognosis of surgery alone in esophageal cancer, multimodality treatments have become popular [2, 3]. There are conflicts about the adequacy of the AJCC Staging System in patients who received chemoradiotherapy (CRT) as neoadjuvant [4]. In the provocative study of Swisher et al., it was stated that pathological complete response to CRT before surgery was an independent prognostic factor for survival and they also proposed a revision of the 6th AJCC Staging System for esophageal cancer [4]. In that study, the authors reported 12 cases whose primary tumour sites were free from malignant cells, while lymph nodes (N1) were positive. The authors suggested “stage 2A” for such cases (ypT0, N1, M0, P0). But there has been no change in the 7th AJCC Staging System for esophageal cancer in that direction. In that case, what should we do? With the case report below, we would like to complicate the situation to some degree by presenting an esophageal cancer CRT-treated patient whose pathologic report was ypT0, N2, M0.

## 2. Case

**Figure 1:**
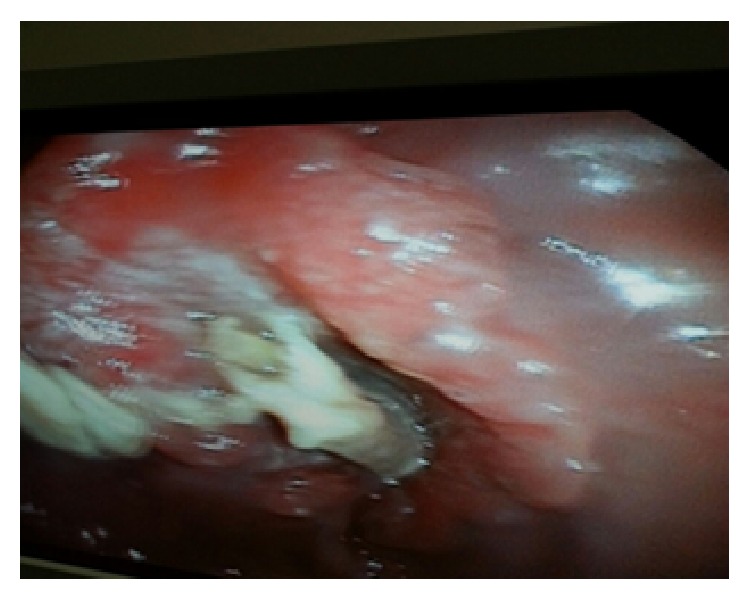
Upper endoscopy of patient showing an ulcerative lesion located between 25 and 38 cm of the esophagus.

**Figure 2:**
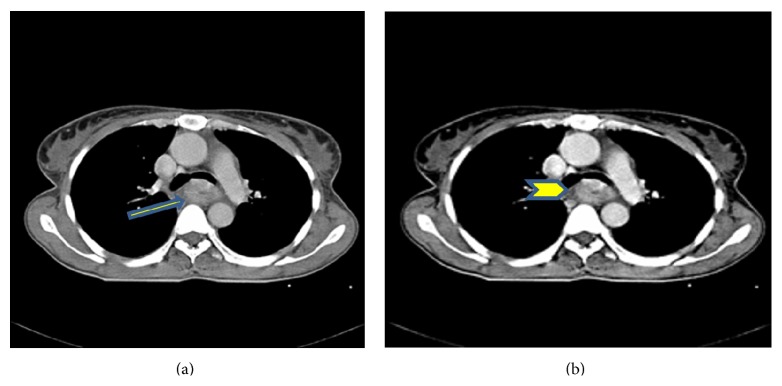
Computed tomography of esophageal mass (arrows) before neoadjuvant therapy.

A 40-year-old female complaining of dysphagia came to our gastroenterology outpatient clinic. In her upper endoscopy, an ulcerative mass was found between 25 and 38 cm from the incisors (Figure 1). After initial work-up and also after the pathology report of an endoscopic biopsy (which was squamous cell cancer), the case was discussed in the oncologic committee. All blood tests (biochemistry, complete blood count) were in the normal range. Pretreatment computed tomography (CT) was as seen in Figure 2. Positron emission tomography (PET) was performed for occult distant metastases and there were no metastases. The patient's performance status was good and she had no comorbidities. Unfortunately, there is no endoscopic ultrasound unit (EUS) in our hospital so this modality was not performed.

**Figure 3:**
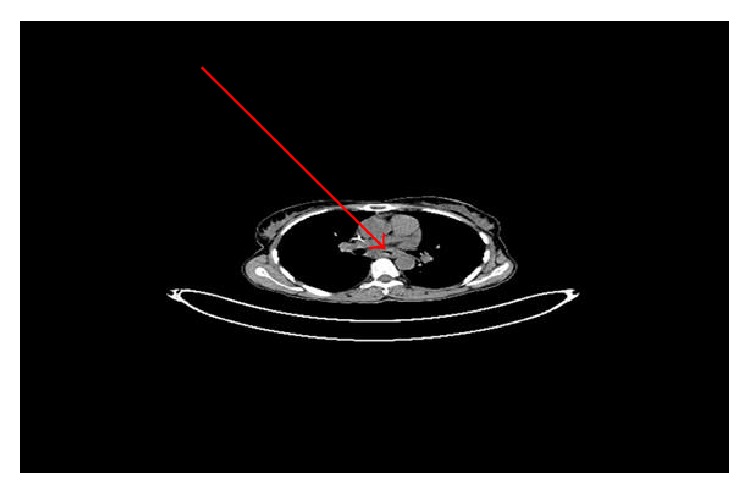
After neoadjuvant therapy, control CT of regressed mass (arrow).

After our case was discussed in the oncologic committee that gathered every week on Thursday, it was decided to characterize the clinical stage of the tumour as T3 or T4a, NX, M0 according to the 7th AJCC Staging System. As expected, for this advanced esophageal tumor, a neoadjuvant CRT decision was taken. In our oncology clinic, chemotherapy and simultaneous radiotherapy were given as follows: Paclitaxel (50 mg/m^2^), Carboplatin (target AUC = 2 mg/Ml·min) plus radiotherapy (41.4 Gy in 23 fractions for 5 days per week). This chemotherapy protocol was given on the first day of radiotherapy and once a week until reaching 5 weeks. After the patient had completed the whole CRT protocol, a control CT was performed (Figure 3). According to the CT report, there was more than 50% response.

Six weeks after CRT, with a curative intention, minimal invasive surgery (MIS) was performed (thoracoscopic esophageal mobilization done first and then gastric mobilization and tube formation performed with laparotomy and cervical anastomosis). There was no tumour or any other lesion when the specimen was examined intraoperatively. No intraoperative complications occurred. However, on the 5th postoperative day, elevated temperature (38.7°C) and tachycardia (145 beats/min) developed. In radiological examination and with methylene blue test, we found a leak in the stapler line on the gastric wall that was formed while creating a gastric tube. Subsequently, despite all interventions and reoperation she was still in the intensive care unit and was intubated at the time of this writing.

**Figure 4:**
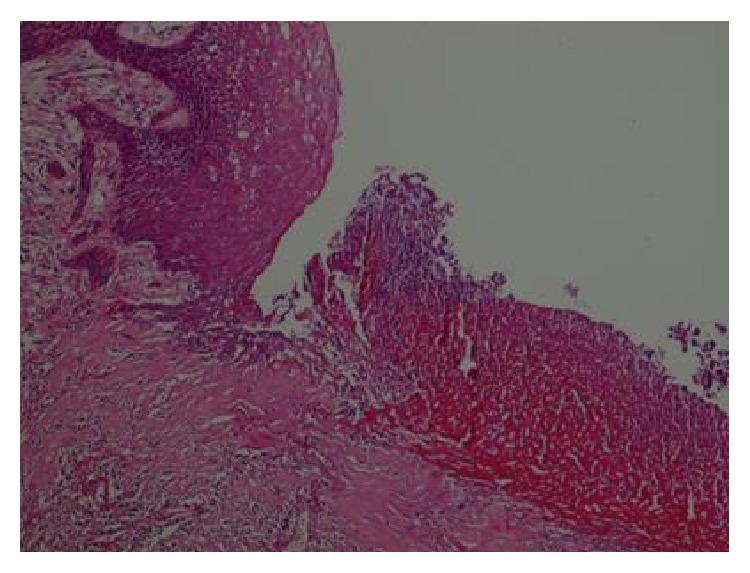
In the upper-left part, there is normal, well-preserved esophageal mucosa, while in the lower-right region there is an ulcer field disrupting the mucosa (H&E stain, ×100).

**Figure 5:**
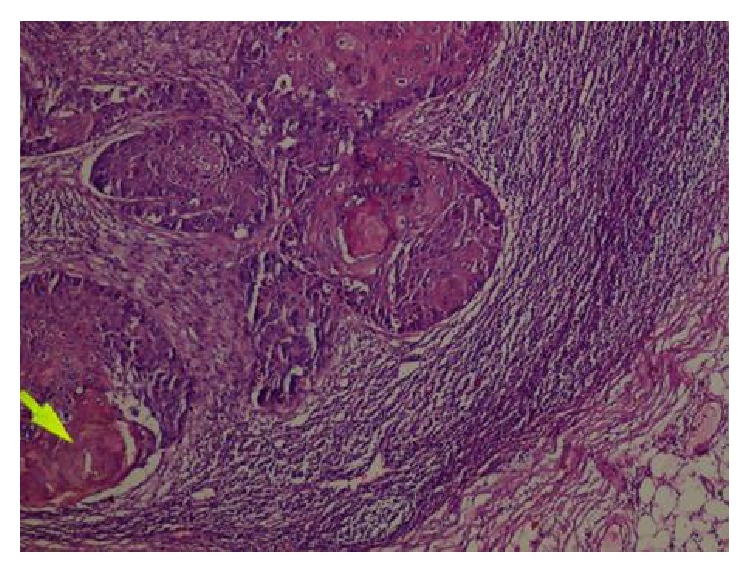
In the lower-right part, a well-preserved lymph node with its peripheral adipose tissue is seen but in the upper-left and lower-left (arrow shows central keratinization) parts of the same lymph node there are squamous cell cancer foci (neoplastic cells with large nuclei, wide eosinophilic cytoplasmic cells).

The pathology report was as follows. “There were no neoplastic cells in the suspected area of the esophageal mucosa upon examination with HMWCK, P63, PAS, and dPAS staining. There were areas of ulceration and severe chronic inflammation and fibrosis in the esophageal lumen: perineural invasion (−), lymphovascular invasion (−). There was no cancer at resection margins. Four metastatic lymph nodes (Figures 4 and 5) were infiltrated with squamous cell cancer. The other 21 harvested lymph nodes were reactive.” We then consulted with the pathologist and requested reevaluation of the specimen and of course the lymph nodes. Nevertheless, the pathological reevaluation was the same.

## 3. Discussion and Conclusion

Interesting proposals about the stage of this case were suggested when the case was discussed in the oncologic committee. One of the proposals was as follows. “It should be evaluated as “ypT4a or T3 and N2, M0” since before CRT the first stage was at this level and after CRT the tumour was present in four lymph nodes, so nothing changed.” Another suggestion was as follows. “Although the pathologists have decided that the esophagus was free from tumour, I believe there are occult cancer foci that could not be examined, so it should be considered as ypTis, N2, M0.”

If it would be decided to evaluate such cases in accordance with the National Comprehensive Cancer Network (NCCN) Guideline version 2.2012, which uses the AJCC Staging System, the part entitled “ESOPH-6” should be considered [5]. In that part, “Observe” is suggested for squamous cell cancer patients who received CRT and were node positive. The clinician would start to “observe” the patient with four metastatic lymph nodes.

If Swisher et al. were called for help, they would suggest that “…the group of patients treated with CRT who are found to have a complete pathologic response at the primary but involved nodes in the specimen (ypT0, N1, M0) should be classified a favourable stage 2 prognosis or a stage 2A…” [4]. Interestingly, all patients reported in the above-mentioned study had 1 or 2 positive (N1) lymph nodes, while in our case report there were 4 (N2) positive lymph nodes. If we think in this way, we must accept our own case as an advanced stage, for example, between stages 3A and stage 3C.

In the study conducted by Rizk et al., which analysed patients with esophageal adenocarcinoma who received CRT before esophagectomy, it was shown that involved lymph nodes and metastatic disease were the best predictors of survival and that depth of invasion and degree of treatment response were less predictive [6]. We think there is a similar tendency in the squamous cell cancer type of esophageal cancer as well. We also believe that although complete pathological response may be seen in post-CRT esophageal specimens, the number of retrieved lymph nodes and the number of metastatic lymph nodes are more important for later treatment and follow-up plans.

Someone can ask, although the exact advantages and disadvantages of CRT have not yet been demonstrated completely, how can we consider the response of tumour to CRT as a prognostic criterion? We think there is huge confusion about multimodality therapies and about the consequences of those therapies. As we have shown by this case report, there is still no consensus about prognosis and staging of patients who have received CRT.

Consequently, despite the growing use of neoadjuvant treatment in locally advanced esophageal cancer in our institute, we do not have a protocol for the evaluation of these patients' pathology reports. We believe that new studies and new ideas are needed to resolve this dilemma associated with CRT.

## References

[1] Rice T. W., Rusch V. W., Ishwaran H., Blackstone E. H. (2010). Cancer of the esophagus and esophagogastric junction: data-driven staging for the seventh edition of the American Joint Committee on Cancer/International Union Against Cancer Cancer Staging Manuals. *Cancer*.

[2] Medical Research Council Oesophageal Cancer Working Party. Writing Committee, Bancewicz J., Clark P. I., Smith D. B., Donnelly R. J. (2002). Surgical resection with or without preoperative chemotherapy in oesophageal cancer: a randomised controlled trial. *The Lancet*.

[3] Urschel J. D., Vasan H. (2003). A meta-analysis of randomized controlled trials that compared neoadjuvant chemoradiation and surgery to surgery alone for resectable esophageal cancer. *The American Journal of Surgery*.

[4] Swisher S. G., Hofstetter W., Wu T. T. (2005). Proposed revision of the esophageal cancer staging system to accommodate pathologic response (pP) following preoperative chemoradiation (CRT). *Annals of Surgery*.

[5] http://www.nccn.org/professionals/physician_gls/pdf/esophageal.pdf.

[6] Rizk N. P., Venkatraman E., Bains M. S. (2007). American Joint Committee on Cancer staging system does not accurately predict survival in patients receiving multimodality therapy for esophageal adenocarcinoma. *Journal of Clinical Oncology*.

